# Delayed recall memory impairment in patients with Parkinson's disease

**DOI:** 10.1590/S1980-5764-2016DN1003006

**Published:** 2016

**Authors:** Arthur Oscar Schelp, Cristiane Lara Mendes-Chiloff, Vanessa Cristina Paduan, José Eduardo Corrente, Fabrício Diniz de Lima, Juliana Cristine Nunes Marchette, Gustavo José Luvizuto, Rodrigo Bazan

**Affiliations:** 1 Universidade Estadual Paulista, Faculdade de Medicina Botucatu, Departamento de Neurologia, Psicologia and Psiquiatria, Botucatu SP, Brazil.; 2 Serviço de Psicologia, Hospital das Clínicas da Faculdade de Medicina de Botucatu, PS, Brazil.; 3 Universidade Estadual Paulista, Instituto de Biociências, Departamento de Estatística, Botucatu SP, Brazil.; 4 Residente de Neurologia, Faculdade de Medicina Botucatu, SP, Brazil.; 5 Serviço de Reabilitação, Hospital das Clinicas da Faculdade de Medicina de Botucatu, SP, Brazil.

**Keywords:** Parkinson's disease, dementia, ageing

## Abstract

Age is one of the risk factors for dementia in patients with Parkinson's disease (PDD). Distinct cognitive syndromes of Parkinson's disease (PD) have been identified in previous studies. Questions about the role of such cognitive disorders in PD outcomes, especially memory dysfunction, in patients with PD remain unanswered.

**Objective:**

To establish possible correlations between delayed recall memory (episodic memory), age, and other demographic variables in patients with PD.

**Methods:**

A two-stage protocol was applied. Patients with delayed recall memory compromise, selected based on a brief battery of tests (BBRC-Edu), were classified as dementia cases and submitted to the Mattis Dementia Rating Scale (MDRS). Data from patients with memory disturbances were compared against individuals without episodic memory impairment, and correlated with age and demographic variables.

**Results:**

Except for identification and naming, all subtests in the screening battery showed a significant difference (p≤0.0001) between the memory-compromised group (case) and the group without memory impairment (no case). The results also correlated negatively with age (p≤0.0001) and positively with level of education (p=0.0874) in patients with PD.

**Conclusion:**

The analysis showed a significant relationship between age and dementia characterized by impaired episodic memory. The findings support reports of a wide spectrum of neuropsychological performance impairment in PD with age, particularly dementia associated with memory deterioration. No correlations between disease duration and cognitive dysfunction were evident.

## RESUMO

Idade é um fator de risco bem determinado para surgimento de demência em pacientes com doença de Parkinson (PDD). Estudo prévio tem demonstrado diferentes distúrbios cognitivos em portadores da doença de Parkinson (PD). O significado do comprometimento da memória é pouco compreendido.

**Objetivo:**

Estabelecer possíveis correlações entre comprometimento de memória de evocação tardia (memória episódica), idade e outras variáveis demográficas em pacientes com DP.

**Métodos:**

Os pacientes foram submetidos a um protocolo dividido em duas etapas. Os pacientes com alterações na memória de evocação tardia (memória episódica), selecionados a partir de bateria breve de rastreio cognitivo (BBRC-Edu) foram classificados como portadores de demência e submetidos ao exame de rastreio para demência de Mattis (MDRS). Os dados relacionados a comprometimento da memória, foram comparados ao grupo que não apresentava alterações e correlacionados a idade e outras variáveis demográficas.

**Resultados:**

Com exceção de identificação e nominação, todos os sub-testes mostraram diferenças significativas (p≤0,0001) entre o grupo que apresentava disfunções na memória episódica e o grupo que não apresentava alterações. Foi demonstrada correlação negativa com idade (p≤0,0001) e positiva com nível educacional (p=0.0874).

**Conclusão:**

O estudo registrou correlação significativa entre idade e demência caracterizada por comprometimento da memória episódica. Os resultados dão suporte para existência de amplo espectro de disfunções associadas ao envelhecimento, em pacientes com PD. Não foram demonstradas correlações significativas com tempo de doença.

## INTRODUCTION

Increasing patient life expectancies call for a greater understanding of the influence of age on the different dysfunctions that occur over the course of degenerative diseases. Ageing is one of the well-defined risk factors for the incidence of dementia in patients with Parkinson's disease (PD).^1^ ^,^ ^2^ PD could itself be a result of ageing.^3^ Some cohort series show an incidence for dementia with PD (PDD) of over 65%, with the relative risk being 5.1 times that of controls.^4^ ^,^ ^5^ In patients with PD, advancing age is associated with worsening of motor symptoms and a lowering of response to medical treatment.^6^ The relationship between motor symptoms and cognitive deficits in PD is also well established.^7^ ^,^ ^8^ The broad spectrum of cognitive disorders observed in different phases of the development of PD,^9^ including cognitive patterns associated with Lewy Body Dementia (LBD) and Alzheimer's disease (AD), supports the hypothesis that other distinct pathologies may be superimposed on symptoms associated with PDD.^10^ ^,^ ^11^ Some evidence supports the existence of a greater prevalence of non-synuclein-related symptoms with advancing age.^12^ Compromised episodic memory is a hallmark symptom of AD and has a well-demonstrated association with ageing.^13^ The identification of particular forms of cognitive deterioration may contribute to the understanding of the relationship among the dementia spectrum, ageing, and PD. The aim of the present study was to analyse the association between dementia associated with delayed recall memory dysfunction in PD patients and demographic variables.

## METHODS

Study design and procedure. A cross-sectional observational study was carried out over a 2-year period in patients diagnosed with PD and being treated at the Outpatient Unit for Movement Disorders at the Clinicas Hospital of Botucatu Medical School, Sao Paulo, Brazil. The Research Ethics Committee of Botucatu Medical School, São Paulo State University, approved the study. All participants, or their legal guardians, provided written informed consent.

A two-stage protocol was applied. Initially, the patients were evaluated using the Brief Cognitive Battery (BBRC-Edu)^14^ (screening tests for detecting the presence of cognitive impairment). Subsequently, those patients in whom memory impairment was identified were evaluated by a specific instrument, the Mattis Dementia Rating Scale (MDRS), validated for the Brazilian population.^15^ The BBRC-Edu included tests of visual perception (naming of ten simple drawings), incidental and immediate memory, learning, verbal fluency, delayed recall (after 5 min), and recognition tasks (recognition of ten familiar figures presented among new ones). Specialized psychologists applied the test. If the patients were in the ON or extreme OFF phase, the tests were repeated at another date and time, and only this second evaluation was included in the analysis. The patients with episodic memory compromise (delayed recall with cut-off score ≤7), were classified as instances of dementia. The MDRS includes five subscales of distinct cognitive domains: attention, initiative/perseverance, construction, conceptualization and memory, with a cut-off score of 122. Socio-demographic (sex, age, and formal education level) and clinical history (diagnosis and treatment time) data were collected.

Selection and description of participants.Over a period of two consecutive years, 125 patients were selected randomly for inclusion in the study. The inclusion criteria were: subjects under supervised care for at least 1 year with a diagnosis of PD, and aged over 18 years. The exclusion criteria were a prior history of stroke, atypical PD, vitamin B12 deficiency, or metabolic encephalopathy.

Statistical analysis. Although in this observational cross-sectional study causal relationships could not be evaluated, the variables were classified as: i) predictive variables, ii) confounding variables, and iii) outcomes. We estimated the possible effects of double and triple interactions between variables on the occurrence of dementia.

Descriptive measures were obtained for quantitative and qualitative variables, and a comparison was made between groups (case and non-case) using Student's *t*-test. Considering episodic memory as a response variable, a regression model was fitted with age, educational level and time since diagnosis as explanatory variables. All analyses were carried out using Statistical Analysis Software (SAS) for Windows v.9.3. The significance level was set at 5% for the tests applied.

## RESULTS

Among the 125 patients studied, 12 did not finish the proposed analysis, giving a total of 113 patients, of which 63.6% were male. The mean age of the sample was 68.09±9.43 years and the mean formal education level was 3.91±3.23 years. The mean length of time since the patients developed PD was 7.67±5.22 years. Tables 1 and 2 depict patient socio-demographic data and results on the screening battery of sub-tests. A significant difference was observed between the group of patient not exhibiting compromised memory on the screening subtests (no case) and the group exhibiting impairment (case). This difference was in relation to age (p≤0.001) and for scores on all screening battery subtests (p≤0.001), except for the identification/naming subtest (Table 2). Tables 3 and 4 show the correlations of performance with age and formal education. Thus, age was negatively correlated with performance on the scale (p=0.0094) whereas formal education was positively correlated (p=0.0797) both with the applied screening test and the MDRS. Table 5 shows the age correlation with the MDRS subscales. No correlation with disease duration was found (Tables 3 and 4).

**Table 1:** Descriptive measures of socio-demographic variables and performance on sub-tests.

Variables		Mean±SD	N	Minimum	Maximum	Median
Socio-demographic variables	Age	68.09±9.43	113	42.00	83.00	70.00
	Formal education (years)	3.91±3.23	93	0.00	15.00	4.00
	Time since diagnosis	7.67±5.22	111	1.00	23.00	6.00
Performance on screening sub-tests	Identification/Naming	9.71±1.26	110	1.00	10.00	10.00
	Incidental memory	5.14±1.80	110	0.00	9.00	5.00
	Immediate memory	6.55±2.32	110	0.00	10.00	7.00
	Learning	7.27±2.32	110	0.00	10.00	8.00
	Delayed recall	6.15±2.82	110	0.00	10.00	7.00
	Recognition	8.35±2.71	110	0.00	10.00	9.50

SD: standard deviation.

**Table 2:** Comparison between groups (occurrence and non-occurrence of dementia).

Variables		Non-occurrence (n=67)	Occurrence (n=46)	p-value*
Socio-demographic variables	Age	64.94±9.68	72.67±6.90	<0.0001
	Formal education (years)	4.38±3.42	3.32±2.92	0.0874
	Time since diagnosis	7.45±4.98	7.91±5.40	0.7692
Performance on screening sub-tests	Identification/naming	9.75±1.23	9,64±1.32	0.3760
	Incidental memory	5.69±1.52	4.33±1.88	≤0.0001
	Immediate memory	7.49±1.96	5.20±2.15	≤0.0001
	Learning	8.37±1.76	5.69±2.11	≤0.0001
	Delayed recall	7.63±2.04	4.02±2.41	≤0.0001
	Recognition	9.42±1.41	6.80±3.35	≤0.0001

*t-test for independent samples.

**Table 3:** Model of multiple linear regression for performance on BBRC-Edu (dependent variable - 5 min. recall memory).

Variables	Estimate	Std. Error	p-value
Intercept	14.19824	3.74899	0.0005
Age	-0.13981	0.05100	0.0094
Formal education (years)	0.20501	0.11375	0.0797
Time since diagnosis	-0.07230	0.06131	0.2459

**Table 4:** Model of multiple linear regression for performance on MDRS.

Variables	Estimate	Std. Error	p-value
Intercept	208.10242	37.74367	<.0.0001
Age	-1.57871	0.51344	0.0039
Formal education (years)	2.58489	1.14519	0.0300
Time since diagnosis	-0.69516	0.61728	0.2673

**Table 5:** Spearman Correlation between age and cognitive impairment.

Mattis Sub-tests						
	Attention	Initiation and perseveration	Construction	Conceptualization	Memory	Total
r	-0.41	-0.50	-0.50	-0.37	-0.35	-0.50
p	0.014	0.002	0.002	0.028	0.038	0.002

## DISCUSSION

The socio-demographic characteristics of the sample studied (Table 1) are similar to those of the general elderly population in Brazil,^16^ except for the predominance of males in the group. This could be explained by the fact that PD is more prevalent in men of advanced age.^17^ Formal education is relevant to the present study, since it was associated with worse results on the subtests (Table 2). As has been observed elsewhere,^18^ formal education is markedly deficient in Brazil.

Some studies call attention to the heterogeneous pattern of cognitive dysfunction in patients newly diagnosed with PD.^19^ The question of whether there is a type of dementia specifically associated with PD remains a matter of debate. Although it is difficult to differentiate LBD and AD in PDD, it is nonetheless accepted that each presents a distinct pattern of cognitive impairment.^11^ ^,^ ^12^

The screening test used to identify patients affected by episodic memory dysfunction includes evaluation of incidental, immediate memory, delayed recall and recognition. The brief battery of tests shows good specificity and sensitivity when used in populations with a low level of education.^20^ Tests of delayed recall memory are highly accurate detectors of dementia in AD.^21^ On the other hand, immediate recall, as measured by word-list tests, is less accurate in distinguishing patients with very mild AD from normal healthy controls.^22^ The use of pictures to detect disturbances in delayed recall is still regarded as the gold standard for assessing memory in a wide range of aged individuals.^23^

The analysis revealed a significant relationship between age and a dementia that involves compromised episodic memory, as determined by the screening test (Table 2). With the exception of visual perception and naming, all other memory assessments showed deficits (p≤0.0001). The Mattis evaluation (Tables 4 and 5) confirmed the results of the screening test, showing compromise across all domains. The age correlation indicates that the older the patient, the lower the Mattis sub-test scores, reflecting a homogeneous worsening of cognitive skills with aging in PD demented patients. 

The demonstration of a relationship between dementia and ageing in patients with PD corroborate the results of other studies.^1^ ^,^ ^4^ ^,^ ^5^ An earlier study reported a wide spectrum of impairments in neuropsychological function emerging with ageing, including, in particular, dementia associated with memory dysfunction.^2^ In the cited report, patients with late onset Parkinsonism performed worse than those with early onset in all memory functions, particularly in picture completion and associative learning (Wechsler memory scale I). Another study failed to find differences in memory impairment among an early and late-onset group, compared with age-matched controls^24^ and questioned the possibility of concomitant AD pathology. However, the fact that episodic memory dysfunction is significantly correlated with aging in patients with PD reinforces the hypothesis that dementia involving memory deterioration during PD could result from concurrent AD. The existence of two cognitive syndromes of PD was outlined by a previous cohort study;^25^ PD may involve two distinct cognitive syndromes that evolve independently, with the dementia related to an increase in tau protein transcription, and effects on dopaminergic structures associated with frontal-executive disorders. Dementia was diagnosed in patients with a Mini-Mental State Examination (MMSE) score of ≤2.^27^ After 7.9 years of follow-up, the data showed a persistent strong statistical correlation between ageing and impaired performance on a task of verbal and semantic fluency.^26^ Since the study defined dementia using a verbal task, the disruption should be considered one of semantic rather than episodic memory. On the other hand, a meta-analysis of verbal fluency deficits in patients with PD failed to demonstrate any qualitative difference between demented and non-demented patients.^27^

The test of visual perception and naming showed no marked differences between the memory-compromised and unimpaired groups (Tables 2 and 3). As stated in a recent review,^28^ visual perception may be less intrinsically related to learning than to reward-related phenomena like motivation and attention. It is reasonable to suppose that when a learning test was given, motivation and attention were higher than in the initial moments of neuropsychological evaluations that demonstrated memory disturbances. Identification and naming can be influenced by semantic memory and other cognitive functions, and do not specifically evaluate episodic memory. It should also be remembered that this was a cross-sectional study, including individuals at different stages of the disease, with various kinds of disturbed memory function, including problems with working and semantic memory. The role of distinct cerebral structures in the incidence and progression of PD dementia remains a matter of debate. The demonstration that only axial impairment was correlated with age, whereas dementia incidence was associated with bradykinesia and speech, suggests that both dopaminergic and non-dopaminergic structures are involved in the etiopathogenesis of dementia in PD.^7^ Hornykiewicz et al., in 1984,^29^ reported that concurrent cortical noradrenaline and cholinergic function deficiencies, as well as basal ganglia dopamine deficiency are key factors in the development of dementia in PD. The indications that dopaminergic structures may be associated with frontal-executive disorders that develop independently,^25^ offer clues to distinguishing different forms of cognitive deterioration in PD. To understand the phenotypic diversity of dementia associated with PD, it is crucial to consider the possibility of subgroups, identified by pathogenesis and aetiology.^30^ This is reasonable considering the varied cognitive disabilities that appear during the course of PD. Although recent studies indicate that at least two distinct forms of cognitive dysfunction arise as PD progresses, the current study shows no evidence of distinct sub-groups suffering from a compromise of frontal or anterior cortical function. Caution must be exercised in interpreting our results. A cross-sectional study minimizes the impact of the data collected. On the other hand, it provides additional evidence supporting the hypothesis that dementia in PD, when initially associated with memory disturbances, develops in distinct ways that worsen with advancing age.

The present study, focused on cognitive deterioration, corroborates the hypothesis that at least one distinctive form of cognitive impairment can be attributed to PD. This data should be validated with longitudinal studies and larger samples including other variables. The novel on-going studies investigating the molecular genetics and pathogenesis of PD, should shed new light on how to identify distinct subgroups of patients, each of which may require a specific therapeutic approach and prognosis.
